# Chronic inflammatory demyelinating polyneuropathy-like neuropathy as an initial presentation of Crohn’s disease

**DOI:** 10.1186/s12883-015-0302-8

**Published:** 2015-03-28

**Authors:** Suji Kim, Seok-Jae Kang, Ki-Wook Oh, Byung Kyu Ahn, Hang Lak Lee, Dong Soo Han, Kiseok Jang, Young Seo Kim

**Affiliations:** Department of Radiology, Kangbuk Samsung Medical Center, Seoul, Republic of Korea; Department of Neurology, College of Medicine, Hanyang University, 17 Haengdang-dong, Seongdong-gu, Seoul 133-792 Republic of Korea; Department of General Surgery, College of Medicine, Hanyang University, Seoul, Republic of Korea; Department of Gastroenterology, College of Medicine, Hanyang University, Seoul, Republic of Korea; Department of Pathology, College of Medicine, Hanyang University, Seoul, Republic of Korea

**Keywords:** Chronic inflammatory demyelinating polyneuropathy, Crohn’s disease

## Abstract

**Background:**

Chronic inflammatory demyelinating polyneuropathy (CIDP) is a rare complication of Crohn's disease (CD), and it is uncertain whether it is associated with CD itself or with its treatment. We describe a case of CIDP-like neuropathy as an initial symptom of CD. The neurologic symptoms of the patient which responded partially to intravenous immunoglobulin (IVIG) recovered after resection of the appendiceal CD.

**Case presentation:**

A 17-year-old male had experienced three separate attacks of motor weakness and paresthesia of all four extremities over a period of 7 months. The electrophysiologic findings revealed a demyelinating sensory-motor polyneuropathy which was compatible with CIDP. However, repeated intravenous IVIG (2 g/kg) treatment gave only a partial response. Four days after the last discharge, he was diagnosed as appendiceal CD after surgical resection of a periappendiceal abscess. His neurologic symptoms and electrophysiologic findings recovered without any maintenance therapy.

**Conclusions:**

CIDP-like neuropathy can be an initial presentation of CD, and recovery of the CIDP symptoms may result from resection of the CD. Clinicians should be aware of the possibility of CD in patients with intractable CIDP symptoms.

## Background

Crohn's disease (CD) is a relapsing, transmural inflammatory disease of the gastrointestinal mucosa that can affect the entire gastrointestinal tract form mouth to anus [1]. It is considered a systemic disease because extraintestinal manifestations such as uveitis, arthritis, pleuritis, myocarditis, primary sclerosing cholangitis, pancreatitis, ankylosing spondylitis and tendinitis can develop [1]. Neurologic manifestations can also occur, the most common being myelopathy, arterial stroke, myopathy, multiple sclerosis and epilepsy [2-4]. Peripheral neuropathy is also reported to be a neurologic complication, and it can be associated with immune-mediated inflammation, micronutrient deficiencies (e.g., vitamin B12, vitamin D, copper) and iatrogenic causes (e.g., metronidazole, TNF-α antagonists) [3,5-8]. A few cases of chronic inflammatory demyelinating polyneuropathy (CIDP) have been reported [7-11], but in those cases the patients developed their symptoms after CD had been established during various treatments, making it difficult to determine if they were caused by the disease or were treatment-related. Here, we describe a CIDP-like neuropathy patient who had 3 episodes of motor weakness as the initial presentation of concealed appendiceal CD. The neurological symptoms and abnormal electrophysiologic findings gradually improved after surgical resection of the appendiceal CD, without CIDP treatment.

## Case presentation

**Figure 1:**
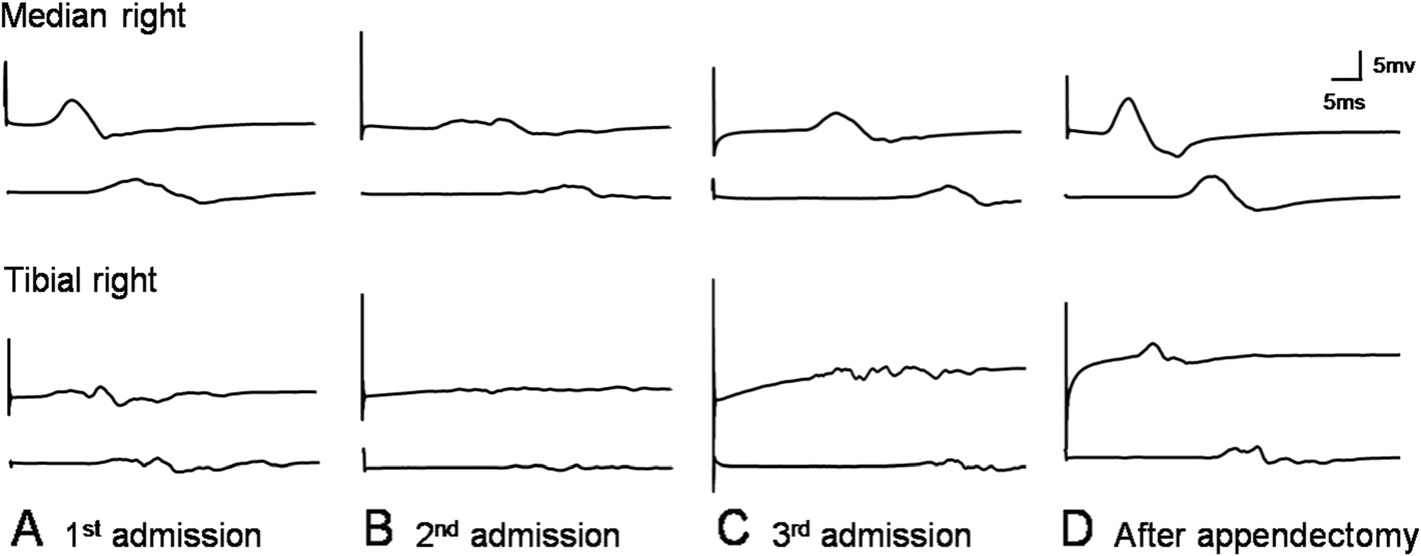
**Serial changes of compound motor action potentials (CMAPs) of the median and tibial nerves. (A)** The first attack before the first intravenous immunoglobulin, **(B)** the second attack 8 weeks after the first attack, **(C)** the third attack 4 months after the second treatment, and **(D)** 6 months after appendectomy without immuno-modulating therapy.

A 17-year-old male without any medical history was admitted with ascending weakness of both lower extremities that had progressed for about two weeks. He had upper respiratory infection one week ago. At the time of admission, his upper respiratory infection was improved and physical examinations in neck, chest and abdomen were unremarkable. On neurologic examination, he had symmetric weakness of both lower extremities (MRC grade 4) with paresthesia of both hands and feet. Deep tendon reflexes were absent in the upper and lower extremities. Routine laboratory findings, including serum complete blood count, biochemistry, urine analysis and C-reactive protein, were normal. Cerebrospinal fluid (CSF) protein level was elevated at 72 mg/dL without pleocytosis and immunoglobulin G levels in serum (3370 mg/dL) and CSF (13.8 mg/dL) were increased. Extensive laboratory investigations including, thyroid function, anti-nuclear antibodies, anti-ganglioside, myelin-associated glycoprotein and viral markers, were all negative. Motor nerve conduction study (NCS) of the median, ulnar, tibial and common peroneal nerves showed prolonged motor terminal latencies and F-wave latencies, and decreased compound muscle action potential (CMAP) amplitudes, sensory nerve action potential (SNAP) amplitude and nerve conduction velocity (NCV) (Table 1). Conduction block and temporal dispersion were also seen in the NCS, compatible with demyelinating polyneuropathy (Figure 1A). With the impression of AIDP, we started intravenous immunoglobulin (0.4 g/kg per day) for 5 days and the patient was discharged after subjective improvement of neurological symptoms.

**Table 1:** **Serial nerve conduction study findings**

**Nerve**	**1st admission (2012.5.15)**	**2nd admission (2012.7.19)**	**3rd admission (2012.11.28)**	**After appendectomy (2013.6.18)**
Median motor, right				
CMAP amplitude (mV)	6.1	**2.5**	**4.6**	5.1
Distal latency (m/s)	**4.92**	**11.4**	**15.3**	**5.8**
NCV (m/s)	**32.2**	**16.9**	**14.5**	**20.7**
F-wave latency (m/s)	**40.1**	**33.4**	**Absent**	**61.9**
Ulnar motor, right				
CMAP amplitude (mV)	9.4	6	**3.8**	5.3
Distal latency (m/s)	**3.27**	**8.4**	**9.89**	**4.8**
NCV (m/s)	**32.7**	**21.3**	**12**	**17.8**
F-wave latency (m/s)	**33.6**	**41.4**	**Absent**	**64**
Peroneal motor, right				
CMAP amplitude (mV)	**0.62**	**No response**	**No response**	**0.37**
distal latency (m/s)	**9.43**	**No response**	**No response**	**9.9**
NCV (m/s)	**27.3**	**No response**	**No response**	**17.7**
F-wave latency (m/s)	**80.4**	**Absent**	**Absent**	**Absent**
Tibial motor, right				
CMAP amplitude (mV)	**3.0**	**0.56**	**0.38**	**2.1**
distal latency (m/s)	**5.63**	**13.6**	**29.9**	**9.1**
NCV (m/s)	49.6	46.9	**35.8**	**26.3**
F-wave latency (m/s)	**60.2**	49.3	**Absent**	**83.6**
Median sensory, right				
SNAP amplitude (mV)	**No response**	**No response**	**No response**	**No response**
NCV (m/s)	**No response**	**No response**	**No response**	**No response**
Sural sensory, right				
SNAP amplitude (μV)	17.8	**No response**	**No response**	**No response**
NCV (m/s)	**17.9**	**No response**	**No response**	**No response**

Abnormal values are shown in bold.

CMAP: compound muscle action potential, SNAP: sensory nerve action potential, NCV: nerve conduction velocity.

Eight weeks after the first onset of symptoms, he complained of further progressive motor weakness and tingling sensations in the four extremities. Electrophysiologic studies showed that the demyelinating sensory-motor polyneuropathy had worsened since the previous study (Table 1, Figure 1B). Guillain-Barré syndrome with treatment-related fluctuations or acute-onset CIDP was considered, and intravenous immunoglobulin (0.4 g/kg per day) was administered for 5 days, again with clinical improvement of symptoms.

Four months after the second event, the patient complained of recurrent progressive motor weakness and sensory changes. Repeated NCS showed no evoked responses in the median, ulnar, tibial and common peroneal nerves, which was compatible with severe demyelination (Table 1, Figure 1C). With the impression of CIDP, immunoglobulin (0.4 g/kg per day) was administered for 5 days and there was only slight improvement of symptoms.

**Figure 2:**
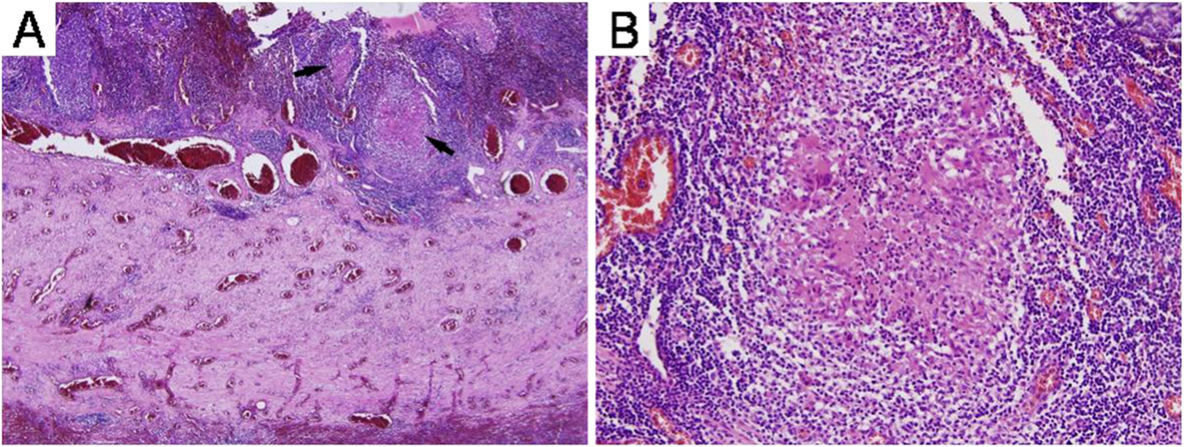
**Pathologic findings of Crohn’s disease involving the appendix.** The appendiceal wall is diffusely thickened by transmural lymphocytic infiltration and fibrosis, with scattered granulomas (**A**, arrows, H&E, original magnification x40). A large, well-formed, non-necrotizing granuloma is present in the mucosa (**B**, H&E, original magnification x200).

Four days after the last discharge, the patient visited the emergency department on account of newly developed severe pain in the right lower abdominal quadrant with fever. He had no gastrointestinal symptoms, including diarrhea, prior to development of the abdominal pain. Physical examination of the abdomen revealed tenderness and rebound tenderness on the right lower quadrant. Abdominal computed tomography (CT) prompted suspicion of a periappendiceal abscess, and emergent surgical resection of the ileum, cecum and appendix was performed. Appendiceal biopsy revealed diffuse wall thickening due to transmural lymphocytic infiltration and fibrosis, with scattered non-necrotizing granulomas, which was compatible with Crohn’s disease (Figure 2). Since it was localized to the appendix and subsequent gastrofiberscopy and colonofiberscopy were unremarkable, he was diagnosed with appendiceal Crohn's disease. After resection of the appendiceal Crohn's disease his motor weakness and the sensory change in all four limbs gradually recovered without any maintenance treatment. Motor nerve conduction study after seven months showed improvement of electrophysiologic parameters and waveforms (Table 1, Figure 1D). After 12 months of appendectomy, he only showed minimal motor and sensory deficits on extremities and he was able to run. Also there are no gastrointestinal symptoms including diarrhea after surgery until that time.

## Conclusions

We report the first case in which CIDP-like neuropathy was the initial presentation of concealed CD. We may assume that the CIDP-like neuropathy was the initial symptom of CD since recurrent and intractable neurological symptoms preceded the diagnosis of CD, and the symptoms recovered without maintenance therapy after surgical resection of the appendiceal CD.

The origin of CIDP is still unclear, but it is widely accepted that it is an autoimmune disease with underlying immunopathology involving autoreactive T cells and B cells [12]. Because CIDP has an autoimmune basis, it can occur in association with diseases such as HIV infection and hepatitis C, Sjogren's syndrome, monoclonal gammopathy of unknown significance, melanoma, lymphoma, diabetes and inflammatory bowel diseases [12]. Only 8 cases of CIDP associated with CD have been reported and we summarized in the Table 2 when clinical information were available [7-11,13]. Of those, 5 cases had already been diagnosed as CD 1 to 30 years before CIDP symptoms developed [7-9]. Since nutritional deficiency or treatment-related neurotoxicity could have occurred during those periods, it is unclear whether the CIDP was caused by the CD itself or by other mechanisms.

**Table 2:** **Cases of CIDP associated with Crohn's disease**

**Reference**	**Age/Sex**	**IBD activity**	**Gap**	**IBD treatment**	**CIDP treatment**
our case	18/M	CA	−7 months	none	IVIG
6	60/F	RR	30 years	TNF-α antagonist (infliximab)	IVIG, prednisolone, azathioprine
7	49/M	Q	20 years	none	IVIG, plasmapheresis, fludarabine, methotrexate
7	47/F	RR	1 years	etanercept, prednisolone	IVIG, prednisolone, plasmapheresis
7	50/M	RR	23 years	azathioprine	IVIG, prednisolone
8	36/M	CA	4 years	mesalazine, metronidazole, ciprofloxacin, azathioprine	Plasmapheresis, prednisolone
9	32/M	CA	0 year	none (newly diagnosed)	IVIG, prednisolone, azathioprine
10	63/F	CA	0 year	none (newly diagnosed)	IVIG, prednisolone

CA, currently active; Q, quiescent; RR, remitting/relapsing; UK, unkown; IVIg, intravenous immunoglobulin.

In the two other cases the CIDP and CD occurred at the same time, but the patients had only one acute progressive symptom, which was not differentiated from acute inflammatory demyelinating polyradiculoneuropathy (AIDP) [10,11]. In addition, they had abdominal symptoms including diarrhea and rectal hemorrhage. Our patient suffered recurrent motor weakness and sensory changes on three occasions over a 7 month period. He also had elevated CSF protein and typical demyelinating-type nerve-conduction findings. Although we did not perform a nerve biopsy and did not confirmed same T-cells from appendix, CSF and nerve, we were able to diagnose the patient as CIDP. The most important and unique finding in this case was that his sensory and motor symptoms developed gradually over 7 months, in very close temporal association to the onset of IBD symptoms. Considering that Crohn’s disease is not an acute disease with sudden onset, extra-intestinal immunologic effects due to the disease itself may have led to recurrent CIDP like symptoms. Considering that Crohn's disease is not an acute disease with sudden onset, subclinical immunologic effects due to the disease itself may lead to recurrent CIDP symptoms. Therefore, we would cautiously suggest that we should consider inflammatory bowel disease as a hidden possible concurrent or preceding disease when a patient has recurrent episodes of CIDP.

Another noteworthy finding in our patient is that he recovered from the CIDP symptoms after resection of the appendiceal CD. Although 3 times of IVIG treatment could have influenced the clinical course of the patient, there was only minimal responses before removal of the periappendiceal abscess. Furthermore, the symptoms and electrophysiological findings gradually improved without any treatment for CIDP. Based on our patient’s clinical courses, we believe that surgical resection may have more pronounce effect on clinical stabilization of the patient. CD confined to the appendix occurs in younger patients and is known to be less aggressive and have a low recurrence rate [14,15]. It can also be the primary or sole manifestation of the disease. Since, appendiceal biopsy of our patient revealed non-caseous granulomas, and colonofiberscopy showed no granulomatous lesions, he was diagnosed as appendiceal CD. Considering our patient’s clinical course, it is possible that the appendiceal CD may have influenced the CIDP-like neuropathy. Appendix is known as an immunological organ, and it may have variable effects on immunologic diseases such as idiopathic inflammatory bowel disease and CIDP-like neuropathy. In this case, CIDP-like neuropathy symptoms were stabilized after surgical resection of the appendiceal CD. We suggest that common immunologic mechanism could be present in those two diseases and non-aggressive limited disease may develop distant neurological symptoms by immunologic mechanisms.

In summary, we describe a case of CIDP as an initial presentation of CD, with recovery of the CIDP symptoms after resection of the appendiceal CD. We suggest that inflammatory bowel disease should be considered a possible underlying disease when investigating patients with CIDP.

### Consent

Written informed consent was obtained from the patient for publication of this Case report and any accompanying images. A copy of the written consent is available for review by the Editor of this journal.

## Abbreviations

CIDP: Chronic inflammatory demyelinating polyneuropathy
CD: Crohn’s disease
IVIG: Intravenous immunoglobulin
CSF: Cerebrospinal fluid
NCS: Nerve conduction study
CMAP: Compound muscle action potential
SNAP: Sensory nerve action potential
NCV: Nerve conduction velocity
AIDP: Acute inflammatory demyelinating polyradiculoneuropathy
